# Layered alkali propano­ates *M* ^+^(C_2_H_5_COO)^−^; *M* ^+^ = Na^+^, K^+^, Rb^+^, Cs^+^

**DOI:** 10.1107/S2056989020011469

**Published:** 2020-08-28

**Authors:** Jan Fábry, Erika Samolová

**Affiliations:** a Inst. of Physics of the Czech Academy of Sciences, Na Slovance 2, 182 21 Praha 8, Czech Republic

**Keywords:** crystal structure, hydrogen bonding, carboxyl­ates, the Cambridge Structural Database, positional disorder, occupational disorder

## Abstract

The title alkali propano­ates poly[(μ_5_-propano­ato)alkali(I)], *M* ^+^(C_2_H_5_COO)^−^, with alkali/*M* ^+^ = Na^+^, K^+^, Rb^+^ and Cs^+^, show close structural similarity, which is manifested by the coordination of the cations by six oxygen atoms in a chessboard motif, forming a bilayer. This bilayer is situated between hydro­phobic layers composed of dangling ethyl chains from the carboxyl­ate groups. Stacking of these two-dimensional sandwiches, which are parallel to (001), forms the title structures. Each metal cation is coordinated by six O atoms in the form of a distorted trigonal prism. One pair of these oxygen atoms belongs to a bridging, bidentately coordinating carboxyl­ate anion, while each of the other four oxygen atoms belongs to different carboxyl­ate groups, which are in a bridging monodentate mode. Despite the close similarity, each of the studied alkali propano­ates crystallizes in a different space group. The atoms are in general positions, except for the cation in K^+^(C_2_H_5_COO)^−^, which is situated on a mirror plane. Positional disorder of the methyl groups that are disordered over two positions is present in the Na^+^ and K^+^ propano­ates, in contrast to the Rb^+^ and Cs^+^ propano­ates. In the Na^+^ compound, the occupational parameters of the disordered methyl groups are different compared to the K^+^ compound where they are equal. This difference results in doubling of the *a* unit-cell parameter of the Na^+^ compound with respect to that of the K^+^ compound, otherwise the structures are homeotypic. In Cs^+^ propano­ate, a disorder of the methyl H atoms is observed.

## Abstract 2

The structures of four alkali propionates, *M* ^+^(C_2_H_5_COO)^−^; *M* ^+^ = Na^+^, K^+^, Rb^+^, Cs^+^, have been determined. All of them show close structural similarity, which is manifested by the coordination of the cation by six oxygen atoms in a chessboard motif, forming a bilayer. This bilayer is situated between hydro­phobic layers composed of dangling ethyl chains. The structures are built up by stacking of these sandwiches.

## Chemical context   

The structures of the alkali propano­ates, *M* ^+^(C_3_H_5_O_2_)^−^, with exception of Li^+^(C_3_H_5_O_2_)^−^ (Martínez Casado *et al.*, 2009 ▸), have not been determined so far, despite their assumed simplicity. The structure of the chemically related compound Tl^+^(C_3_H_5_O_2_)^−^ was determined by Martínez Casado *et al.* (2010 ▸).
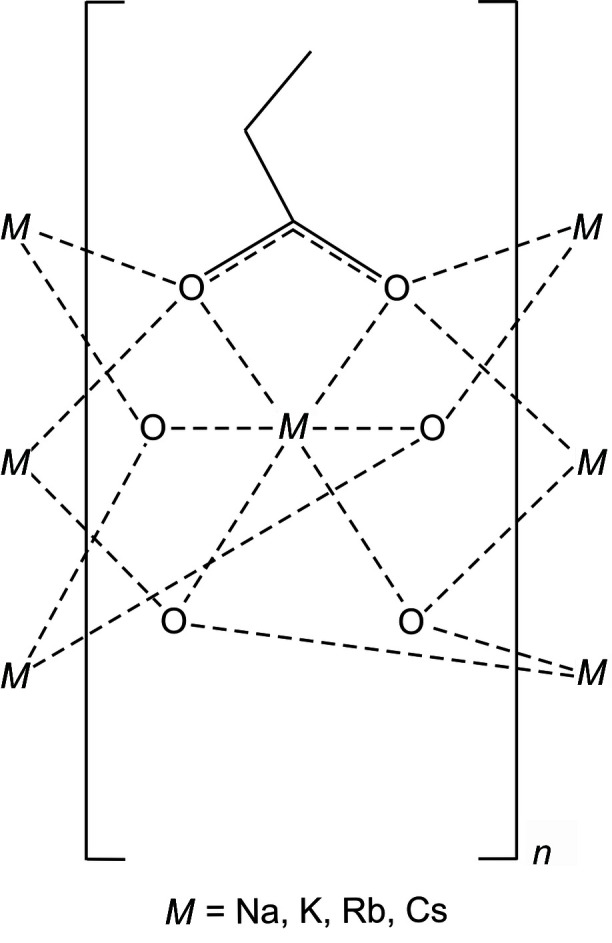


On the other hand, the physical properties of some alkali propano­ates, together with related alkanoates, have been studied. Phase transitions were studied in alkali propano­ates together with alkali formates, acetates and butyrates by Ferloni *et al.* (1975 ▸) employing differential scanning calorimetry. The lowest-temperature phase transitions in Li, Na, K, Rb and Cs propano­ates take place at 533, 470, 258 (±2), 317 (±2) and 314 K, respectively.

Cingolani *et al.* (1979 ▸) determined the phase-transition temperatures in Li^+^(C_3_H_5_O_2_)^−^, Na^+^(C_3_H_5_O_2_)^−^ and K^+^(C_3_H_5_O_2_)^−^ by conductometric measurements. The determined phase-transition temperatures corresponded well with those reported by Ferloni *et al.* (1975 ▸), except for Li^+^(C_3_H_5_O_2_)^−^ where the phase transition was detected at 553 K. Martínez Casado *et al.* (2009 ▸) determined the phase-transition temperature for the Li compound at 549.1 (±0.7) K in the virgin sample. The temperature of this phase transition varied during repeated cooling and heating.

The unit-cell parameters of the title structures have been determined in the past. In addition, Martínez Casado *et al.* (2009 ▸) determined the unit-cell parameters of lithium propano­ate by single-crystal X-ray diffraction at 100, 160 and 298 K. Massarotti & Spinolo (1979 ▸) determined the unit-cell parameters for three phases of sodium propano­ate by powder X-ray diffraction. Entry No. 00-042-1901 in the powder diffraction file (PDF-4; Gates-Rector & Blanton, 2019 ▸) is derived from the latter powder data collection at 298 K. Cingolani *et al.* (1979 ▸) determined the unit-cell parameters for three phases of sodium propano­ate and for phases I and II of potassium propano­ate by powder X-ray diffraction but not for the lowest-temperature existing phase III of the latter compound. Massarotti & Spinolo (1980 ▸) determined the unit-cell parameters for phases I and II of potassium propano­ate but not for the lowest-temperature existing phase III either. Entries No. 00-042-1856–00-042-1859 in PDF-4 (Gates-Rector & Blanton, 2019 ▸) are derived from the data collection of the latter authors. Massarotti & Spinolo (1980 ▸) also determined two phases of Rb propano­ate by powder X-ray diffraction above 317 K, but not phase III existing below this temperature.

In the present study, the title structures were determined at 240 K, *i.e.* in the stability region of the known lowest-temperature phases. This means that the temperature regions in which the phases III of K^+^(C_3_H_5_O_2_)^−^ and Rb^+^(C_3_H_5_O_2_)^−^ (Cingolani *et al.*, 1979 ▸) exist have been measured. However, the lattice parameters of K^+^(C_3_H_5_O_2_)^−^ reported here are in a fair agreement with the lattice parameters of phase II of K^+^(C_3_H_5_O_2_)^−^, which exists between 258 (±2)–352.5 (±0.6) K (Ferloni *et al.*, 1975 ▸; Cingolani *et al.*, 1979 ▸; Massarotti & Spinolo, 1980 ▸). The same holds for the lattice parameters of Rb^+^(C_3_H_5_O_2_)^−^ and phase II of Rb^+^(C_3_H_5_O_2_)^−^, which is reported to exist between 317 (±2) and 564 K by Ferloni *et al.* (1975 ▸) and Massarotti & Spinolo (1980 ▸), respectively. The reported unit cell of Rb^+^(C_3_H_5_O_2_)^−^ has all the inter­axial angles equal to 90°, in contrast to the present study. PDF-4 entries 00-032-1982–00-032-1984 (Gates-Rector & Blanton, 2019 ▸) are based on the experiments carried out by Massarotti & Spinolo (1980 ▸).

No match regarding caesium propano­ate has been found in the Cambridge Structural Database (Groom *et al.*, 2016 ▸; version 5.41 from November 2019); however, there is an entry (No. 00-049-2031 in PDF-4; Gates-Rector & Blanton, 2019 ▸) that is attributed to this compound. The corresponding unit-cell volume *V* = 1355.38 Å^3^ is close to that observed recently at room temperature in Cs^+^(C_2_H_5_COO)^−^·H_2_O with *V* = 1334.25 (4) Å^3^ (Samolová & Fábry, 2020 ▸). Therefore, it can not be excluded that the reported phase in PDF-4 is in fact a hydrate. It should be emphasized that for each particular compound, their reported unit-cell parameters correspond to each other while multiplication of the unit-cell volume takes place in some cases.

Pretransitional phenomena have been observed in some of the title structures, which indicates a complicated structural rearrangement taking place before melting [see also the study of Li^+^(C_3_H_5_O_2_)^−^, Na^+^(C_3_H_5_O_2_)^−^ and K^+^(C_3_H_5_O_2_)^−^ by Cingolani *et al.* (1979 ▸), and the study of Li^+^(C_3_H_5_O_2_)^−^ by Martínez Casado *et al.* (2009 ▸)]. Such phenomena are more prominent in the structures with longer hydro­phobic chains, *e.g.* in butyrates (Duruz & Ubbelohde, 1972 ▸).

It should be mentioned that the crystals in the current study were cooled down instantly from room temperature to 240 K by putting them into a stream of a cooling gas. On the other hand, the measurement was carried out at temperatures not far from the thermodynamic equilibrium in which the room-temperature-grown crystals are assumed to exist. Our experience has shown that cooling down the crystals to very low temperatures does not necessarily mean a better resolution or better quality of the measured data.

An important structural feature of alkali alkanoates *M* ^+^C_*n*_H_2*n*+1_COO^−^ (*n* > 2) seems to be their layered arrangement. For example, a layered structure has been observed in Li(C_3_H_5_O_2_) (Martínez Casado *et al.*, 2009 ▸) as well as in Li_2_Cd(C_2_H_5_COO)_4_ (Griffith & Amma, 1992 ▸), despite the fact that Li^+^ is coordinated by four oxygen atoms in contrast to the six oxygens in the title structures. On the other hand, the layered structure of Tl(C_3_H_5_O_2_) (Martínez Casado *et al.*, 2010 ▸) is more complicated because it contains three independent Tl^+^ cations. Two of them (Tl1 and Tl3) are situated in a similar coordination to that in the title structures while Tl2 is situated in a roughly octa­hedral coordination. The presence of more than one symmetry-independent Tl^+^ cation even in simple structures is quite common. This is the case, for example, in a high-temperature phase of Tl_2_MoO_4_ (Friese *et al.*, 1999 ▸) or in Tl_2_WO_4_ (Okada *et al.*, 1979 ▸) where three unique cations are present. Another example of a layered structure where the metal–oxygen sheet is surrounded by hydro­phobic organic layers is potassium palmitate KC_16_H_31_O_2_ (Dumbleton & Lomer, 1965 ▸).

## Structural commentary   

**Figure 1:**
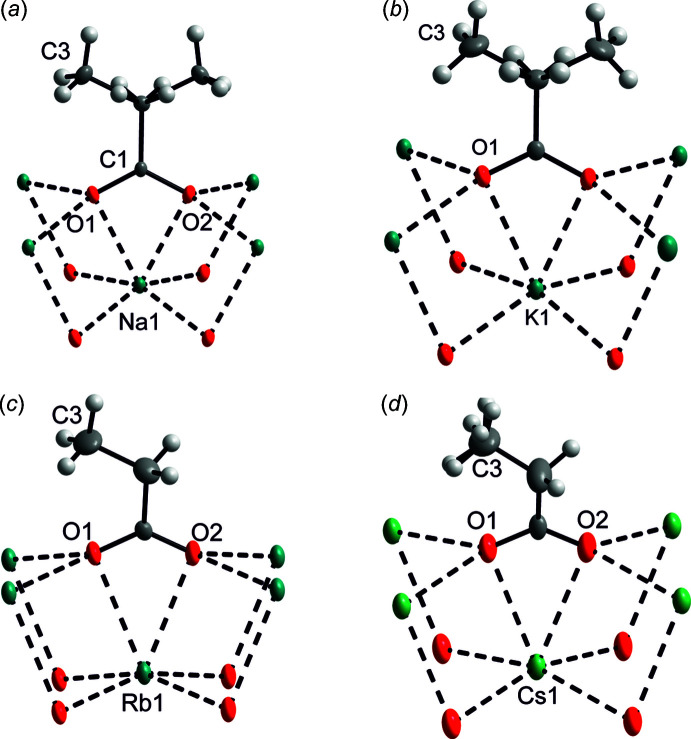
The common structural motifs in the title structures: (*a*) Na^+^(C_3_H_5_O_2_)^−^, (*b*) K^+^(C_3_H_5_O_2_)^−^, (*c*) Rb^+^(C_3_H_5_O_2_)^- and^ (*d*) Cs^+^(C_3_H_5_O_2_)^−^. Displacement ellipsoids are shown at the 30% probability level. The cations, O, C and H atoms are shown as green, red, grey ellipsoids and as tiny light-grey spheres, respectively.

Fig. 1 ▸ *a*–*d* show the coordination environments around the central cations, which are situated in general positions except for K^+^(C_2_H_5_COO)^−^ where the cation is on a mirror plane (Wyckoff position 2 *e)*. The central cations are surrounded by six oxygens with two of them stemming from the same carboxyl­ate group. The cation–oxygen distances are different; expectedly, the distances from the pair of oxygen atoms belonging to the same carboxyl­ate group are the longest. In the title structures, the angles O_carboxyl­ate_—*M*—O_carboxyl­ate_ decrease monotonously in the series *M* = Na, K, Rb, Cs: 52.39 (5), 45.92 (5), 43.93 (10), 41.28 (8)°. The bond-valence sums (Brese & O’Keeffe, 1991 ▸) of the cations are 1.062 (2), 1.156 (3), 1.109 (5) and 1.042 (4) valence units for the Na, K, Rb, Cs compounds, respectively. The motifs shown in Fig. 1 ▸ *a*–*d* are quite similar to those observed in potassium acrylate and potassium methacrylate (Heyman *et al.*, 2020 ▸) while the unit-cell parameters of the latter compounds also show a close correspondence to those of the title structures.

**Figure 2:**
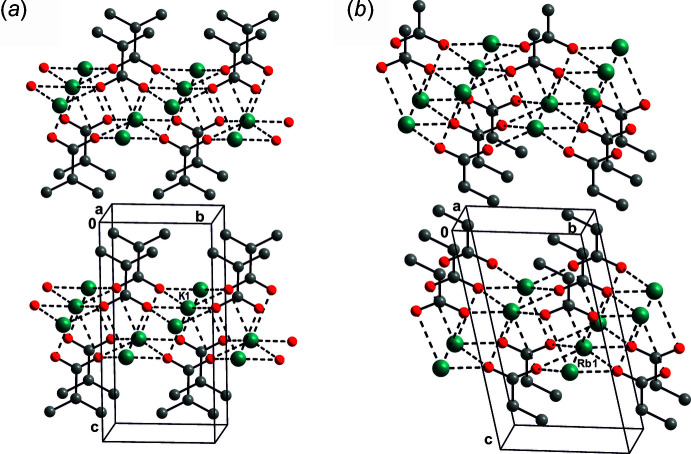
The crystal packing of (*a*) K^+^(C_3_H_5_O_2_)- and (*b*) Rb^+^(C_3_H_5_O_2_)^−^. K or Rb, O and C atoms are shown as green, red and grey spheres, respectively. H atoms are omitted for clarity.

The common prominent feature of the title structures is the presence of an oxygen–metal bilayer that is sandwiched by ethyl chains. The layers are aligned parallel to (001), and packing of these layers forms the title structures. The structural motifs in all of the title structures are quite similar, and the structures can be considered as homeotypic (Lima de Faria *et al.*, 1989 ▸). Because of their similarity, overall packing views are given only for the potassium and rubidium propano­ates (Fig. 2 ▸ *a*,*b*) because they represent the structures with positionally disordered and ordered methyl groups, respectively. The unit-cell parameters of the depicted structures are of similar size in contrast to the other structures.

The positional disorder observed in the Na and K propano­ates (not in Rb and Cs propano­ates) is worth being discussed in detail. Table 1 ▸ lists the distances between neighbouring carbon atoms of the methyl­ene and methyl groups. In Rb^+^(C_3_H_5_O_2_)^−^ and Cs^+^(C_3_H_5_O_2_)^−^, these distances are larger than in Na^+^(C_3_H_5_O_2_)^−^ and K^+^(C_3_H_5_O_2_)^−^. This means that shorter distances between the methyl groups seem to be correlated with the observed positional disorder of the methyl groups. The disordered methyl groups are situated in rows, which are aligned parallel to the *b* axis in Na^+^(C_3_H_5_O_2_)^−^ and K^+^(C_3_H_5_O_2_)^−^. However, the assumed switching by rotation from one disordered position to another should also affect neighbouring rows in the *ab* plane. A correlated ordering of the ethyl groups is thus expected to take place. This situation is analogous to that observed in BaCa_2_(C_3_H_5_O_2_)_6_ where the methyl carbon atoms get as close as 4.05 (2) Å (Stadnicka & Glazer, 1980 ▸). Table 2 ▸ shows that in the case of Na^+^(C_3_H_5_O_2_)^−^ and K^+^(C_3_H_5_O_2_)^−^, the positional disorder can bring these groups even as close as 2.609 (8) and 2.651 (9) Å, respectively, a value that clearly indicates the impossibility of simultaneous occupation of these sites by both groups. This short value also indicates the presence of thermal fluctuations. These fluctuations would provoke revolution of the ethyl chain to the other, *i.e.* the disordered site, while causing a domino effect by forcing the other ethyl chains to revolve in order to remove as much repulsion as possible. The torsion angles O1/O2—C1—C2—C3, which are listed in Table 2 ▸, also throw some light on the observed disorder. They are close to 0 or 180° for the disordered Na and K title compounds in contrast to the ordered Rb and Cs title compounds. The disordered methyl groups are situated in energetically similar or even identical positions in the Na and K compounds, respectively, in contrast to the the Rb and Cs compounds.

In the studied crystal of Na^+^(C_3_H_5_O_2_)^−^, the refined occupational parameters of the disordered methyl group converged to 0.808 (4) for one and 0.192 (4) for the other orientation. A hypothetical structure of Na(C_3_H_5_O_2_) where no positional disorder occurs would be described in a unit cell with a halved unit-cell parameter *a* relative to the title structure. The space group of such a hypothetical structure would be *P*2_1_ instead of *P*2_1_/*a*. [The transformation into the halved unit cell can also be carried out according to equation (1) in section 4]. Halving of the unit-cell parameter *a* would also be caused by a positional disorder in the ratio 0.50:0.50, provided that the blocks with the ordered mol­ecules are sufficiently small. The space-group type of such a hypothetical structure would be *P*2_1_/*m*, which is equal to that of the reported structure of K(C_3_H_5_O_2_). The unit-cell parameter *a* of the title structure Na(C_3_H_5_O_2_) can be halved and transformed into the one that was reported by Massarotti & Spinolo (1979 ▸) or Cingolani *et al.* (1979 ▸) for phase III, the known lowest-temperature existing phase (determined by a powder diffraction study). In other words, it seems that the occupational parameters of the disordered ethyl groups can vary in different crystals of Na^+^(C_3_H_5_O_2_)^−^. More probably, because of the repulsion of the methyl groups, the phases, which had been subjected to powder diffraction experiments, rather correspond to the structures described in the space groups *P*2_1_/*m*.

## Synthesis and crystallization   

The title compounds were prepared by dissolution of the pertinent alkali carbonates with propionic acid in the respective molar ratio of 1:2 in water. The pH of the solution was adjusted to 6–7 by addition of propionic acid. The solutions were filtered and the excessive amount of water was evaporated at 313 K. Prior to crystallization, which started on the surface of the solution, a more viscous layer seemed to develop. This layer was optically isotropic (no extinction in polarized light), in agreement with the observations for Li(C_3_H_5_O_2_), Na(C_3_H_5_O_2_), and K(C_3_H_5_O_2_) (Cingolani *et al.*, 1979 ▸). During the course of the concentration of the solution, crystals also grew at the bottom of the beaker.

For the preparation of Na(C_3_H_5_O_2_), 1.49 g of Na_2_CO_3_ and 1.04 g of propionic acid were used before adjustment of the pH to 6–7 by propionic acid; for the preparation of K(C_3_H_5_O_2_), 1.49 g of K_2_CO_3_·1.5H_2_O and 0.67 g of propionic acid were used before adjustment of the pH to 6–7 by propionic acid; for the preparation of Rb(C_3_H_5_O_2_), 1.50 g of Rb_2_CO_3_ and 0.48 g of propionic acid were used before adjustment of the pH to 6–7 by propionic acid; for the preparation of Cs(C_3_H_5_O_2_), 1.50 g of Cs_2_CO_3_ and 0.34 g of propionic acid were used before adjustment of the pH to 6–7 by propionic acid.

All of the title compounds are hygroscopic. Crystals of Cs(C_3_H_5_O_2_) turned out to be deliquescent, and from the resulting solution the monohydrate Cs(C_2_H_5_COO)·H_2_O crystallized after some time (Samolová & Fábry, 2020 ▸). Rb(C_2_H_5_COO) also turned out to be deliquescent. K(C_2_H_5_COO) was hygroscopic and the hygroscopicity of Na(C_2_H_5_COO) (Massarotti & Spinolo, 1979 ▸) was confirmed as well.

## Refinement   

Crystal data, data collection and structure refinement details are summarized in Table 3 ▸. Methyl hydrogen atoms were constrained: C_meth­yl_—H_meth­yl_ = 0.96 Å while *U*_iso_(H_meth­yl_) = 1.5*U*_eq_(C_meth­yl_). Attached methyl­ene hydrogen atoms were situated at calculated positions and refined under the constraints C_methyl­ene_—H_methyl­ene_ = 0.97 Å and *U*_iso_(H_methyl­ene_) = 1.2*U*_eq_(C_methyl­ene_).

Na(C_3_H_5_O_2_): It turned out that the ethyl groups are disordered over two positions. The occupational parameters of the methyl groups were refined under the constraint that their sum equal unity, resulting in a 0.808 (4): 0.192 (4) ratio for the methyl groups C3 and C3*a*. The large unit cell can be transformed into the small unit cell that corresponds to that of K(C_3_H_5_O_2_) by the transformation [*a*, *b*, *c*]_small_ = [*a*, *b*, *c*]_large_ [1/2 0 1/2 / 0 1 0 / 0 0 1] [equation (1)].

K(C_3_H_5_O_2_): The ethyl groups are disordered over two positions due to the crystal symmetry, with occupancies equal to 1/2. The positions of the methyl hydrogens were discerned from the difference electron-density map.

Rb(C_3_H_5_O_2_): The positions of the methyl hydrogen atoms were discerned from the difference electron-density map.

Cs(C_3_H_5_O_2_): The methyl hydrogen atoms are equally disordered over two positions. The refined value of the Flack parameter [0.10 (9)] and its standard uncertainty did not enable the absolute structure to be determined reliably (Flack & Bernardinelli, 2000 ▸).

## Supplementary Material

Crystal structure: contains datablock(s) global, I, II, III, IV. DOI: 10.1107/S2056989020011469/wm5573sup1.cif

Structure factors: contains datablock(s) I. DOI: 10.1107/S2056989020011469/wm5573Isup2.hkl

Supporting information file. DOI: 10.1107/S2056989020011469/wm5573Isup6.smi

Structure factors: contains datablock(s) II. DOI: 10.1107/S2056989020011469/wm5573IIsup3.hkl

Supporting information file. DOI: 10.1107/S2056989020011469/wm5573IIsup7.smi

Structure factors: contains datablock(s) III. DOI: 10.1107/S2056989020011469/wm5573IIIsup4.hkl

Supporting information file. DOI: 10.1107/S2056989020011469/wm5573IIIsup8.smi

Structure factors: contains datablock(s) IV. DOI: 10.1107/S2056989020011469/wm5573IVsup5.hkl

Supporting information file. DOI: 10.1107/S2056989020011469/wm5573IVsup9.smi

Supporting information file. DOI: 10.1107/S2056989020011469/wm5573Isup10.cml

Supporting information file. DOI: 10.1107/S2056989020011469/wm5573IIsup11.cml

Supporting information file. DOI: 10.1107/S2056989020011469/wm5573IIIsup12.cml

Supporting information file. DOI: 10.1107/S2056989020011469/wm5573IVsup13.cml

CCDC references: 2024659, 2024658, 2024657, 2024656

Additional supporting information:  crystallographic information; 3D view; checkCIF report

## Figures and Tables

**Table 1:** C_methyl­ene_—C_methyl­ene_, C_methyl­ene_—C_meth­yl_ and C_meth­yl_—C_meth­yl_ distances (Å) in the title structures Atoms C2 and C3 correspond to the methyl­ene and methyl atoms, respectively. The note ’disordered’ indicates that the second atom belongs to the methyl carbon in a disordered position. For Na(C_2_H_5_COO), it is a C3*a* atom, for K(C_2_H_5_COO) it is a C3 atom with the symmetry codes x, xi, xii, xiii.

Compound	Inter­action	*d*	Note
Na^+^(C_2_H_5_COO)^−^			
	C2—C2^i^	3.552 (3)	
	C2—C2^ii^	3.552 (3)	
	C2—C3^iii^	3.973 (4)	
	C2—C3^i^	3.994 (4)	
	C2—C3^ii^	3.703 (4)	
	C2—C3*a* ^iv^	3.973 (13)	disordered
	C2—C3*a* ^ii^	3.723 (14)	disordered
	C3—C3^v^	3.981 (4)	
	C3—C3*a* ^vi^	2.609 (8)	disordered
	C3—C3*a* ^iv^	3.079 (14)	disordered
	C3—C3*a* ^i^	3.547 (16)	disordered
	C3—C3*a* ^ii^	3.558 (16)	disordered
			
K^+^(C_2_H_5_COO)^−^			
	C2—C2^vii^	3.907 (6)	
	C2—C2^viii^	3.907 (6)	
	C2—C3^viii^	3.982 (8)	
	C2—C3^ix^	3.993 (7)	
	C2—C3^x^	3.993 (7)	disordered
	C2—C3^xi^	3.982 (8)	disordered
	C3—C3^vii^	3.907 (11)	
	C3—C3^viii^	3.907 (11)	
	C3—C3^x^	2.997 (8)	disordered
	C3—C3^xii^	3.136 (9)	disordered
	C3—C3^xiii^	2.651 (9)	disordered
			
Rb^+^(C_2_H_5_COO)^−^			
	C2—C2^vii^	4.154 (10)	
	C2—C2^viii^	4.154 (10)	
	C2—C2^xiv^	4.250 (8)	
	C2—C3^viii^	4.228 (11)	
	C2—C3^xv^	4.238 (10)	
	C2—C3^xiv^	4.268 (9)	
	C3—C3^vii^	4.154 (13)	
	C3—C3^viii^	4.154 (13)	
	C3—C3^xvi^	4.154 (13)	
	C3—C3^xvi^	3.908 (12)	
	C3—C3^xvii^	4.035 (11)	
			
Cs^+^(C_2_H_5_COO)^−^			
	C2—C2^vii^	4.424 (13)	
	C2—C2^viii^	4.424 (13)	
	C2—C2^xviii^	4.223 (11)	
	C2—C2^xix^	4.223 (11)	
	C2—C3^vii^	4.790 (14)	
	C2—C3^viii^	4.531 (14)	
	C2—C3^xviii^	4.258 (12)	
	C2—C3^xix^	4.114 (12)	
	C3—C3^vii^	4.424 (16)	
	C3—C3^viii^	4.424 (16)	
	C3—C3^xx^	3.882 (13)	
	C3—C3^xviii^	4.634 (13)	
	C3—C3^xxi^	3.882 (13)	
	C3—C3^xix^	4.634 (13)	

Symmetry codes: (i) *x* − 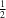, −*y* + 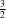, *z*; (ii) *x* + 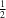, −*y* + 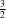, *z*; (iii) −*x* + 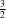, *y* + 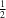, −*z* + 2 (iv) −*x* + 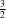, *y* − 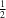, −*z* + 2 (v) −*x* + 2, −*y* + 1, −*z* + 2 (vi) *x*, *y* − 1, *z* (vii) *x* − 1, *y*, *z* (viii) *x* + 1, *y*, *z* (ix) −*x*, *y* + 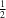, −*z* + 2 (x) −*x*, −*y* + 1, −*z* + 2 (xi) *x* + 1, −*y* + 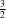, *z* (xii) *x*, −*y* + 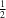, *z* (xiii) *x*, −*y* + 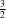, *z* (xiv) −*x* + 2, −*y* + 2, −*z* (xv) −*x* + 1, −*y* + 2, −*z* (xvi) −*x* + 1, −*y* + 1, −*z* (xvii) −*x* + 2, −*y* + 1, −*z* (xviii) *x* − 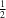, −*y* + 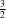, −*z* + 1 (xix) *x* + 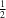, −*y* + 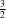, −*z* + 1 (xx) *x* − 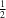, −*y* + 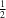, −*z* + 1 (xxi) *x* + 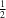, −*y* + 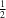, −*z* + 1.

**Table 2:** Torsional angles (°) for the propano­ate fragments in *M* ^+^(C_2_H_5_COO)^−^; *M* ^+^ = Na^+^, K^+^, Rb^+^, Cs^+^

Compound	Atom 1	Atom 2	Atom 3	Atom 4	Angle
Na^+^(C_2_H_5_COO)^−^					
	O1	C1	C2	C3	2.1 (3)
	O2	C1	C2	C3	−178.6 (2)
					
K^+^(C_2_H_5_COO)^−^					
	O1	C1	C2	C3	−0.3 (5)
	O1^xiii^	C1	C2	C3	179.4 (4)
					
Rb^+^(C_2_H_5_COO)^−^					
	O1	C1	C2	C3	−5.1 (8)
	O2	C1	C2	C3	177.0 (5)
					
Cs^+^(C_2_H_5_COO)^−^					
	O1	C1	C2	C3	−16.7 (10)
	O2	C1	C2	C3	165.7 (7)

Symmetry code: (xiii) *x*, −*y* + 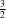, *z*.

**Table 3:** Experimental details

	[Na(C_3_H_5_O_2_)]	[K(C_3_H_5_O_2_)]	[Rb(C_3_H_5_O_2_)]	[Cs(C_3_H_5_O_2_)]
Crystal data				
*M*_r_	96.1	112.2	158.5	206
Crystal system, space group	Monoclinic, *P*2_1_/*a*	Monoclinic, *P*12_1_/*m*1	Triclinic, *P* 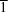	Orthorhombic, *P*2_1_2_1_2_1_
Temperature (K)	240	240	240	240
*a*, *b*, *c* (Å)	7.1048 (4), 5.3003 (3), 11.9035 (7)	3.9070 (17), 5.7872 (17), 11.317 (5)	4.1538 (13), 6.0008 (16), 11.182 (3)	4.4242 (1), 6.2866 (2), 21.4422 (8)
α, β, γ (°)	90, 111.225 (5), 90	90, 94.03 (2), 90	80.038 (10), 81.465 (10), 88.987 (10)	90, 90, 90
*V* (Å^3^)	417.85 (4)	255.25 (18)	271.48 (13)	596.38 (3)
*Z*	4	2	2	4
Radiation type	Cu *K*α	Mo *K*α	Mo *K*α	Mo *K*α
μ (mm^−1^)	1.94	0.90	8.99	6.09
Crystal size (mm)	0.44 × 0.17 × 0.04	0.43 × 0.39 × 0.02	0.32 × 0.28 × 0.03	0.36 × 0.31 × 0.03

Data collection				
Diffractometer	Bruker D8 VENTURE Kappa Duo PHOTON 100 CMOS	Bruker D8 VENTURE Kappa Duo PHOTON 100 CMOS	Bruker D8 VENTURE Kappa Duo PHOTON 100 CMOS	Bruker D8 VENTURE Kappa Duo PHOTON 100 CMOS
Absorption correction	Multi-scan (*SADABS*; Bruker, 2017 ▸)	Multi-scan (*SADABS*; Bruker, 2017 ▸)	Multi-scan (*SADABS*; Bruker, 2017 ▸)	Multi-scan (*SADABS*; Bruker, 2017 ▸)
*T*_min_, *T*_max_	0.484, 0.934	0.700, 0.982	0.160, 0.762	0.218, 0.848
No. of measured, independent and observed [*I* > 3σ(*I*)] reflections	5731, 812, 610	8787, 738, 611	5050, 1568, 963	5255, 1687, 1583
*R*_int_	0.038	0.054	0.069	0.026
(sin θ/λ)_max_ (Å^−1^)	0.619	0.705	0.706	0.703

Refinement				
*R*[*F* > 3σ(*F*)], *wR*(*F*), *S*	0.050, 0.136, 3.60	0.051, 0.087, 2.07	0.047, 0.095, 1.36	0.027, 0.069, 1.63
No. of reflections	812	738	1568	1687
No. of parameters	59	37	55	56
No. of restraints	1	0	0	0
H-atom treatment	H-atom parameters constrained	H-atom parameters constrained	H-atom parameters constrained	H-atom parameters constrained
Δρ_max_, Δρ_min_ (e Å^−3^)	0.48, −0.25	1.41, −0.99	0.88, −0.84	0.64, −0.65

Computer programs: *APEX3* (Bruker, 2017 ▸), *SAINT* (Bruker, 2017 ▸), *SUPERFLIP* (Palatinus & Chapuis, 2007 ▸), *JANA*2006 (Petříček *et al.*, 2014 ▸) and *DIAMOND* (Brandenburg, 2005 ▸).

## supplementary crystallographic information

### Poly[(µ5-propanoato)sodium(I)] (I) Crystal data

**Table:**

[Na(C_3_H_5_O_2_)]	*F*(000) = 200
*M**_r_* = 96.1	There have been used diffractions with I/σ(I)>20 for the unit cell determination.
Monoclinic, *P*2_1_/*a*	*D*_x_ = 1.527 Mg m^−^^3^
Hall symbol: -P 2yab	Cu *K*α radiation, λ = 1.54178 Å
*a* = 7.1048 (4) Å	Cell parameters from 3181 reflections
*b* = 5.3003 (3) Å	θ = 8.0–72.6°
*c* = 11.9035 (7) Å	µ = 1.94 mm^−^^1^
β = 111.225 (5)°	*T* = 240 K
*V* = 417.85 (4) Å^3^	Plate, colourless
*Z* = 4	0.44 × 0.17 × 0.04 mm

### Poly[(µ5-propanoato)sodium(I)] (I) Data collection

**Table:**

Bruker D8 VENTURE Kappa Duo PHOTON 100 CMOS diffractometer	812 independent reflections
Radiation source: IµS micro-focus sealed tube	610 reflections with *I* > 3σ(*I*)
Helios Cu multilayer optic monochromator	*R*_int_ = 0.038
φ and ω scans	θ_max_ = 72.6°, θ_min_ = 8.0°
Absorption correction: multi-scan (*SADABS*; Bruker, 2017)	*h* = −8→8
*T*_min_ = 0.484, *T*_max_ = 0.934	*k* = −6→6
5731 measured reflections	*l* = −14→14

### Poly[(µ5-propanoato)sodium(I)] (I) Refinement

**Table:**

Refinement on *F*^2^	56 constraints
*R*[*F* > 3σ(*F*)] = 0.050	Primary atom site location: charge flipping
*wR*(*F*) = 0.136	H-atom parameters constrained
*S* = 3.60	Weighting scheme based on measured s.u.'s *w* = 1/(σ^2^(*I*) + 0.0004*I*^2^)
812 reflections	(Δ/σ)_max_ = 0.007
59 parameters	Δρ_max_ = 0.48 e Å^−^^3^
1 restraint	Δρ_min_ = −0.25 e Å^−^^3^

### Poly[(µ5-propanoato)sodium(I)] (I) Special details

**Table:**

Refinement. The anisotropic displacement parameters of the disordered methyls were constrained: β11[C3a] = β11[C3], β22[C3a] = β22[C3], β33[C3a] = β33[C3], β12[C3a] = -β12[C3], β13[C3a] = β13[C3], β23[C3a] = -β23[C3]. The positions of the methyl hydrogens were discerned in the difference electron density map. The methyl hydrogens were constrained: Cmethyl—Hmethyl = 0.96?Å while Uiso(Hmethyl) = 1.5Ueq(Cmethyl). The methylene carbons were considered to be superimposed possessing the same positional as well as anisotropic displacement parameters with the overall occupational parameter equal to 1. The distances C2-C3 and C2-C3a were restrained to be equal. The attached methylene hydrogens were situated into the calculated positions with the corresponding occupational parameters and refined under the constraints Cmethylene—Hmethylene = 0.97?Å and Uiso(Hmethylene) = 1.2Ueq(Cmethylene). Their occupancies equalled to those of the occupanices of the pertinent methyl.

### Poly[(µ5-propanoato)sodium(I)] (I) Fractional atomic coordinates and isotropic or equivalent isotropic displacement parameters (Å^2^)

**Table:**

	*x*	*y*	*z*	*U*_iso_*/*U*_eq_	Occ. (<1)
Na1	0.58889 (11)	0.75057 (15)	0.42221 (6)	0.0219 (3)	
O1	0.6929 (2)	0.5409 (3)	0.62475 (11)	0.0257 (6)	
O2	0.6933 (2)	0.9598 (3)	0.62567 (11)	0.0272 (6)	
C1	0.7006 (3)	0.7495 (4)	0.67624 (16)	0.0189 (6)	
C2	0.7176 (3)	0.7518 (4)	0.80791 (18)	0.0252 (7)	
C3	0.7313 (5)	0.4978 (6)	0.8670 (3)	0.0366 (10)	0.808 (4)
C3a	0.732 (2)	1.0057 (14)	0.8673 (11)	0.0366 (10)	0.192 (4)
H1c2	0.832208	0.854412	0.854817	0.0303*	0.808 (4)
H2c2	0.605413	0.845792	0.815159	0.0303*	0.808 (4)
H1c2d	0.606148	0.657665	0.815979	0.0303*	0.192 (4)
H2c2d	0.830804	0.647765	0.855331	0.0303*	0.192 (4)
H1c3	0.83853	0.401966	0.856705	0.0549*	0.808 (4)
H2c3	0.75814	0.520257	0.951449	0.0549*	0.808 (4)
H3c3	0.605919	0.409385	0.83061	0.0549*	0.808 (4)
H1c3a	0.856406	1.085929	0.872783	0.0549*	0.192 (4)
H2c3a	0.620431	1.108883	0.820205	0.0549*	0.192 (4)
H3c3a	0.729143	0.984142	0.946657	0.0549*	0.192 (4)

### Poly[(µ5-propanoato)sodium(I)] (I) Atomic displacement parameters (Å^2^)

**Table:**

	*U*^11^	*U*^22^	*U*^33^	*U*^12^	*U*^13^	*U*^23^
Na1	0.0253 (5)	0.0146 (5)	0.0294 (5)	0.0001 (4)	0.0142 (3)	0.0001 (3)
O1	0.0323 (8)	0.0152 (10)	0.0341 (8)	−0.0011 (6)	0.0174 (7)	−0.0038 (6)
O2	0.0341 (9)	0.0161 (10)	0.0361 (8)	0.0008 (6)	0.0184 (7)	0.0045 (6)
C1	0.0181 (8)	0.0150 (9)	0.0262 (9)	−0.0002 (9)	0.0111 (7)	0.0004 (8)
C2	0.0245 (9)	0.0262 (11)	0.0287 (9)	−0.0003 (11)	0.0141 (7)	−0.0016 (9)
C3	0.0414 (15)	0.0310 (14)	0.0433 (14)	−0.0006 (15)	0.0223 (12)	0.0091 (13)
C3a	0.0414 (15)	0.0310 (14)	0.0433 (14)	0.0006 (15)	0.0223 (12)	−0.0091 (13)

### Poly[(µ5-propanoato)sodium(I)] (I) Geometric parameters (Å, º)

**Table:**

O1—C1	1.255 (3)	C3—H3c3	0.96
O2—C1	1.259 (3)	C3a—H1c3a	0.96
C1—C2	1.528 (3)	C3a—H2c3a	0.96
C2—C3	1.506 (4)	C3a—H3c3a	0.96
C2—C3a	1.506 (9)	Na1—O1	2.5107 (15)
C2—H1c2	0.97	Na1—O1^i^	2.3898 (18)
C2—H2c2	0.97	Na1—O1^ii^	2.4286 (17)
C2—H1c2d	0.97	Na1—O2	2.5190 (15)
C2—H2c2d	0.97	Na1—O2^iii^	2.3944 (19)
C3—H1c3	0.96	Na1—O2^iv^	2.4233 (17)
C3—H2c3	0.96		
			
O1—C1—O2	124.01 (18)	C2—C3a—H3c3a	109.47
O1—C1—C2	118.77 (19)	H1c3a—C3a—H2c3a	109.47
O2—C1—C2	117.21 (19)	H1c3a—C3a—H3c3a	109.47
C1—C2—C3	116.1 (2)	H2c3a—C3a—H3c3a	109.47
C1—C2—C3a	117.1 (5)	O1—Na1—O1^i^	121.28 (5)
C1—C2—H1c2	109.47	O1—Na1—O1^ii^	82.59 (5)
C1—C2—H2c2	109.47	O1—Na1—O2	52.39 (5)
C1—C2—H1c2d	109.47	O1—Na1—O2^iii^	87.29 (5)
C1—C2—H2c2d	109.47	O1—Na1—O2^iv^	115.92 (6)
C3—C2—C3a	126.7 (5)	O1^i^—Na1—O1^ii^	155.01 (5)
C3—C2—H1c2	109.47	O1^i^—Na1—O2	87.20 (6)
C3—C2—H2c2	109.47	O1^i^—Na1—O2^iii^	80.15 (6)
C3a—C2—H1c2d	109.47	O1^i^—Na1—O2^iv^	95.08 (6)
C3a—C2—H2c2d	109.47	O1^ii^—Na1—O2	115.64 (6)
H1c2—C2—H2c2	101.92	O1^ii^—Na1—O2^iii^	94.93 (6)
H1c2d—C2—H2c2d	100.62	O1^ii^—Na1—O2^iv^	78.81 (6)
C2—C3—H1c3	109.47	O2—Na1—O2^iii^	121.43 (5)
C2—C3—H2c3	109.47	O2—Na1—O2^iv^	82.98 (5)
C2—C3—H3c3	109.47	O2^iii^—Na1—O2^iv^	154.50 (5)
H1c3—C3—H2c3	109.47	Na1—O1—Na1^iii^	92.91 (6)
H1c3—C3—H3c3	109.47	Na1—O1—Na1^ii^	97.41 (5)
H2c3—C3—H3c3	109.47	Na1^iii^—O1—Na1^ii^	94.99 (6)
C2—C3a—H1c3a	109.47	Na1—O2—Na1^i^	92.59 (6)
C2—C3a—H2c3a	109.47	Na1—O2—Na1^iv^	97.02 (5)
			
O1—C1—C2—C3	2.1 (3)	O2—C1—C2—C3	−178.6 (2)

Symmetry codes: (i) −*x*+3/2, *y*+1/2, −*z*+1; (ii) −*x*+1, −*y*+1, −*z*+1; (iii) −*x*+3/2, *y*−1/2, −*z*+1; (iv) −*x*+1, −*y*+2, −*z*+1.

### Poly[(µ5-propanoato)potassium(I)] (II) Crystal data

**Table:**

[K(C_3_H_5_O_2_)]	*F*(000) = 116
*M**_r_* = 112.2	*D*_x_ = 1.459 Mg m^−^^3^
Monoclinic, *P*12_1_/*m*1	Mo *K*α radiation, λ = 0.71073 Å
Hall symbol: -P 2yb	Cell parameters from 2906 reflections
*a* = 3.9070 (17) Å	θ = 3.6–30.0°
*b* = 5.7872 (17) Å	µ = 0.90 mm^−^^1^
*c* = 11.317 (5) Å	*T* = 240 K
β = 94.03 (2)°	Plate, colourless
*V* = 255.25 (18) Å^3^	0.43 × 0.39 × 0.02 mm
*Z* = 2	

### Poly[(µ5-propanoato)potassium(I)] (II) Data collection

**Table:**

Bruker D8 VENTURE Kappa Duo PHOTON 100 CMOS diffractometer	738 independent reflections
Radiation source: IµS micro-focus sealed tube	611 reflections with *I* > 3σ(*I*)
Quazar Mo multilayer optic monochromator	*R*_int_ = 0.054
φ and ω scans	θ_max_ = 30.1°, θ_min_ = 3.6°
Absorption correction: multi-scan (*SADABS*; Bruker, 2017)	*h* = −5→5
*T*_min_ = 0.700, *T*_max_ = 0.982	*k* = −8→8
8787 measured reflections	*l* = −15→15

### Poly[(µ5-propanoato)potassium(I)] (II) Refinement

**Table:**

Refinement on *F*^2^	20 constraints
*R*[*F* > 3σ(*F*)] = 0.051	Primary atom site location: charge flip
*wR*(*F*) = 0.087	H-atom parameters constrained
*S* = 2.07	Weighting scheme based on measured s.u.'s *w* = 1/(σ^2^(*I*) + 0.0004*I*^2^)
738 reflections	(Δ/σ)_max_ = 0.002
37 parameters	Δρ_max_ = 1.41 e Å^−^^3^
0 restraints	Δρ_min_ = −0.99 e Å^−^^3^

### Poly[(µ5-propanoato)potassium(I)] (II) Special details

**Table:**

Refinement. There have been discarded the following diffractions for which |Iobs-Icalc|>10σ(Iobs): 4 5 2, 5 1 3, -2 7 3, 5 1 4, 5 2 4, 4 4 4, -2 7 4, 5 1 5, 4 1 9, -2 5 9, -2 6 9, 0 0 11, 2 4 11, 3 0 12, -2 4 13, -2 3 14, -1 2 15

### Poly[(µ5-propanoato)potassium(I)] (II) Fractional atomic coordinates and isotropic or equivalent isotropic displacement parameters (Å^2^)

**Table:**

	*x*	*y*	*z*	*U*_iso_*/*U*_eq_	Occ. (<1)
K1	0.25348 (15)	0.75	0.40739 (6)	0.0273 (2)	
O1	0.2622 (4)	0.5584 (2)	0.63882 (14)	0.0333 (5)	
C1	0.2293 (6)	0.75	0.6884 (3)	0.0255 (9)	
C2	0.1418 (8)	0.75	0.8173 (3)	0.0426 (12)	
C3	0.1000 (18)	0.5210 (11)	0.8750 (5)	0.071 (3)	0.5
H1c2	0.310384	0.840791	0.864035	0.0511*	0.5
H2c2	−0.062022	0.842673	0.825507	0.0511*	0.5
H1c3	0.14797	0.536058	0.959028	0.106*	0.5
H2c3	−0.131148	0.467536	0.858867	0.106*	0.5
H3c3	0.256348	0.411972	0.844314	0.106*	0.5

### Poly[(µ5-propanoato)potassium(I)] (II) Atomic displacement parameters (Å^2^)

**Table:**

	*U*^11^	*U*^22^	*U*^33^	*U*^12^	*U*^13^	*U*^23^
K1	0.0258 (3)	0.0185 (3)	0.0381 (4)	0	0.0057 (2)	0
O1	0.0381 (8)	0.0194 (8)	0.0433 (10)	0.0006 (6)	0.0083 (7)	−0.0024 (6)
C1	0.0175 (12)	0.0246 (15)	0.0346 (18)	0	0.0021 (11)	0
C2	0.0468 (19)	0.045 (2)	0.037 (2)	0	0.0110 (15)	0
C3	0.101 (5)	0.071 (5)	0.043 (4)	−0.003 (4)	0.020 (3)	0.015 (3)

### Poly[(µ5-propanoato)potassium(I)] (II) Geometric parameters (Å, º)

**Table:**

O1—C1	1.253 (2)	C3—H1c3	0.96
C1—C2	1.521 (5)	C3—H2c3	0.96
C2—C3	1.492 (6)	C3—H3c3	0.96
C2—C3^i^	1.492 (6)	K1—O1	2.842 (3)
C2—H1c2	0.97	K1—O1^ii^	2.715 (2)
C2—H1c2^i^	0.97	K1—O1^iii^	2.679 (2)
C2—H2c2	0.97	K1—O1^iv^	2.715 (2)
C2—H2c2^i^	0.97	K1—O1^v^	2.679 (2)
C3—H1c2^i^	1.1598	K1—O1^i^	2.842 (3)
C3—H2c2^i^	1.1347		
			
O1—C1—O1^i^	124.4 (3)	C2—C3—H2c3	109.47
O1—C1—C2	117.80 (14)	C2—C3—H3c3	109.47
O1^i^—C1—C2	117.80 (14)	O1—K1—O1^ii^	113.24 (5)
C1—C2—C3	117.3 (3)	O1—K1—O1^iii^	118.48 (5)
C1—C2—C3^i^	117.3 (3)	O1—K1—O1^iv^	83.21 (5)
C1—C2—H1c2	109.47	O1—K1—O1^v^	87.54 (5)
C1—C2—H1c2^i^	109.47	O1—K1—O1^i^	45.92 (5)
C1—C2—H2c2	109.47	O1^ii^—K1—O1^iii^	92.81 (5)
C1—C2—H2c2^i^	109.47	O1^ii^—K1—O1^iv^	82.22 (5)
C3—C2—C3^i^	125.4 (4)	O1^ii^—K1—O1^v^	157.68 (6)
C3—C2—H1c2	109.47	O1^ii^—K1—O1^i^	83.21 (5)
C3—C2—H1c2^i^	51.01	O1^iii^—K1—O1^iv^	157.68 (6)
C3—C2—H2c2	109.47	O1^iii^—K1—O1^v^	83.55 (5)
C3—C2—H2c2^i^	49.52	O1^iii^—K1—O1^i^	87.54 (5)
C3^i^—C2—H1c2	51.01	O1^iv^—K1—O1^v^	92.81 (5)
C3^i^—C2—H1c2^i^	109.47	O1^iv^—K1—O1^i^	113.24 (5)
C3^i^—C2—H2c2	49.52	O1^v^—K1—O1^i^	118.48 (5)
C3^i^—C2—H2c2^i^	109.47	K1—O1—K1^vi^	96.79 (5)
C3^i^—C2—H1c2^i^	109.47	K1—O1—K1^vii^	92.46 (5)
C3^i^—C2—H2c2^i^	109.47	K1^vi^—O1—K1^vii^	92.81 (5)
C2—C3—H1c3	109.47		
			
O1—C1—C2—C3	−0.3 (5)	O1—C1—C2—C3^i^	−179.4 (4)

Symmetry codes: (i) *x*, −*y*+3/2, *z*; (ii) −*x*, *y*+1/2, −*z*+1; (iii) −*x*+1, *y*+1/2, −*z*+1; (iv) −*x*, −*y*+1, −*z*+1; (v) −*x*+1, −*y*+1, −*z*+1; (vi) −*x*, *y*−1/2, −*z*+1; (vii) −*x*+1, *y*−1/2, −*z*+1.

### Poly[(µ5-propanoato)rubidium(I)] (III) Crystal data

**Table:**

[Rb(C_3_H_5_O_2_)]	*Z* = 2
*M**_r_* = 158.5	*F*(000) = 152
Triclinic, *P*1	There have been used diffraction with I/σ(I)>20
Hall symbol: -P 1	*D*_x_ = 1.939 Mg m^−^^3^
*a* = 4.1538 (13) Å	Mo *K*α radiation, λ = 0.71073 Å
*b* = 6.0008 (16) Å	Cell parameters from 2269 reflections
*c* = 11.182 (3) Å	θ = 4.2–29.0°
α = 80.038 (10)°	µ = 8.99 mm^−^^1^
β = 81.465 (10)°	*T* = 240 K
γ = 88.987 (10)°	Plate, colourless
*V* = 271.48 (13) Å^3^	0.32 × 0.28 × 0.03 mm

### Poly[(µ5-propanoato)rubidium(I)] (III) Data collection

**Table:**

Bruker D8 VENTURE Kappa Duo PHOTON 100 CMOS diffractometer	1568 independent reflections
Radiation source: X-ray tube	963 reflections with *I* > 3σ(*I*)
Quazar Mo multilayer optic monochromator	*R*_int_ = 0.069
φ and ω scans	θ_max_ = 30.1°, θ_min_ = 3.5°
Absorption correction: multi-scan (*SADABS*; Bruker, 2017)	*h* = −5→5
*T*_min_ = 0.160, *T*_max_ = 0.762	*k* = −7→8
5050 measured reflections	*l* = −15→15

### Poly[(µ5-propanoato)rubidium(I)] (III) Refinement

**Table:**

Refinement on *F*^2^	20 constraints
*R*[*F* > 3σ(*F*)] = 0.047	Primary atom site location: charge flipping
*wR*(*F*) = 0.095	H-atom parameters constrained
*S* = 1.36	Weighting scheme based on measured s.u.'s *w* = 1/(σ^2^(*I*) + 0.0004*I*^2^)
1568 reflections	(Δ/σ)_max_ = 0.004
55 parameters	Δρ_max_ = 0.88 e Å^−^^3^
0 restraints	Δρ_min_ = −0.84 e Å^−^^3^

### Poly[(µ5-propanoato)rubidium(I)] (III) Fractional atomic coordinates and isotropic or equivalent isotropic displacement parameters (Å^2^)

**Table:**

	*x*	*y*	*z*	*U*_iso_*/*U*_eq_	
Rb1	0.73165 (12)	0.71678 (9)	0.60411 (5)	0.03307 (18)	
O1	0.7845 (8)	0.6143 (6)	0.3502 (3)	0.0379 (13)	
O2	0.7998 (9)	0.9829 (6)	0.3534 (3)	0.0410 (14)	
C1	0.7720 (11)	0.8167 (9)	0.3016 (4)	0.0285 (17)	
C2	0.7054 (16)	0.8726 (13)	0.1694 (6)	0.063 (3)	
C3	0.689 (2)	0.6740 (14)	0.1052 (6)	0.084 (4)	
H1c2	0.867601	0.979995	0.122034	0.0757*	
H2c2	0.505805	0.958462	0.166536	0.0757*	
H1c3	0.669326	0.727128	0.020563	0.1263*	
H2c3	0.883574	0.586403	0.11031	0.1263*	
H3c3	0.503336	0.581496	0.143673	0.1263*	

### Poly[(µ5-propanoato)rubidium(I)] (III) Atomic displacement parameters (Å^2^)

**Table:**

	*U*^11^	*U*^22^	*U*^33^	*U*^12^	*U*^13^	*U*^23^
Rb1	0.0334 (3)	0.0197 (3)	0.0471 (3)	0.00058 (18)	−0.0087 (2)	−0.00620 (19)
O1	0.048 (2)	0.0192 (19)	0.048 (2)	−0.0005 (17)	−0.0131 (17)	−0.0039 (16)
O2	0.047 (2)	0.022 (2)	0.056 (2)	−0.0019 (18)	−0.0091 (19)	−0.0106 (17)
C1	0.022 (2)	0.027 (3)	0.036 (3)	−0.003 (2)	−0.003 (2)	−0.003 (2)
C2	0.071 (5)	0.062 (5)	0.057 (4)	−0.002 (4)	−0.024 (4)	−0.001 (4)
C3	0.134 (8)	0.079 (6)	0.049 (4)	−0.013 (6)	−0.031 (5)	−0.022 (4)

### Poly[(µ5-propanoato)rubidium(I)] (III) Geometric parameters (Å, º)

**Table:**

Rb1—O1	2.984 (4)	O2—C1	1.253 (7)
Rb1—O1^i^	2.877 (4)	C1—C2	1.523 (8)
Rb1—O1^ii^	2.839 (4)	C2—C3	1.502 (11)
Rb1—O2	2.953 (4)	C2—H1c2	0.97
Rb1—O2^iii^	2.862 (4)	C2—H2c2	0.97
Rb1—O2^iv^	2.820 (4)	C3—H1c3	0.96
Rb1—C1	3.312 (5)	C3—H2c3	0.96
O1—C1	1.245 (6)	C3—H3c3	0.96
			
O1—C1—O2	125.5 (5)	O1—Rb1—O2	43.93 (10)
O1—C1—C2	118.7 (5)	O1—Rb1—O2^iii^	109.85 (11)
O2—C1—C2	115.7 (5)	O1—Rb1—O2^iv^	116.72 (10)
C1—C2—C3	115.7 (6)	O1^i^—Rb1—O1^ii^	93.20 (10)
C1—C2—H1c2	109.47	O1^i^—Rb1—O2	112.60 (11)
C1—C2—H2c2	109.47	O1^i^—Rb1—O2^iii^	82.45 (10)
C3—C2—H1c2	109.47	O1^i^—Rb1—O2^iv^	160.59 (10)
C3—C2—H2c2	109.47	O1^ii^—Rb1—O2	116.19 (10)
H1c2—C2—H2c2	102.39	O1^ii^—Rb1—O2^iii^	160.58 (10)
C2—C3—H1c3	109.47	O1^ii^—Rb1—O2^iv^	83.87 (11)
C2—C3—H2c3	109.47	O2—Rb1—O2^iii^	82.76 (10)
C2—C3—H3c3	109.47	O2—Rb1—O2^iv^	85.67 (11)
H1c3—C3—H2c3	109.47	O2^iii^—Rb1—O2^iv^	93.94 (11)
H1c3—C3—H3c3	109.47	Rb1—O1—Rb1^i^	97.75 (10)
H2c3—C3—H3c3	109.47	Rb1—O1—Rb1^ii^	91.85 (10)
O1—Rb1—O1^i^	82.25 (10)	Rb1—O1—O2	67.30 (14)
O1—Rb1—O1^ii^	88.15 (10)	Rb1^i^—O1—Rb1^ii^	93.20 (10)
			
O1—C1—C2—C3	−5.1 (8)	O2—C1—C2—C3	177.0 (5)

Symmetry codes: (i) −*x*+1, −*y*+1, −*z*+1; (ii) −*x*+2, −*y*+1, −*z*+1; (iii) −*x*+1, −*y*+2, −*z*+1; (iv) −*x*+2, −*y*+2, −*z*+1.

### Poly[(µ5-propanoato)caesium(I)] (IV) Crystal data

**Table:**

[Cs(C_3_H_5_O_2_)]	There have been used diffractions with I/σ(I)>20 for the unit cell determination.
*M**_r_* = 206	*D*_x_ = 2.294 Mg m^−^^3^
Orthorhombic, *P*2_1_2_1_2_1_	Mo *K*α radiation, λ = 0.71073 Å
Hall symbol: P 2ac 2ab	Cell parameters from 5792 reflections
*a* = 4.4242 (1) Å	θ = 3.4–30.0°
*b* = 6.2866 (2) Å	µ = 6.09 mm^−^^1^
*c* = 21.4422 (8) Å	*T* = 240 K
*V* = 596.38 (3) Å^3^	Plate, colourless
*Z* = 4	0.36 × 0.31 × 0.03 mm
*F*(000) = 376	

### Poly[(µ5-propanoato)caesium(I)] (IV) Data collection

**Table:**

Bruker D8 VENTURE Kappa Duo PHOTON 100 CMOS diffractometer	1687 independent reflections
Radiation source: X-ray tube	1583 reflections with *I* > 3σ(*I*)
Quazar Mo multilayer optic monochromator	*R*_int_ = 0.026
φ and ω scans	θ_max_ = 30.0°, θ_min_ = 1.9°
Absorption correction: multi-scan (*SADABS*; Bruker, 2017)	*h* = −6→5
*T*_min_ = 0.218, *T*_max_ = 0.848	*k* = −8→7
5255 measured reflections	*l* = −29→25

### Poly[(µ5-propanoato)caesium(I)] (IV) Refinement

**Table:**

Refinement on *F*^2^	Primary atom site location: charge flipping
*R*[*F* > 3σ(*F*)] = 0.027	H-atom parameters constrained
*wR*(*F*) = 0.069	Weighting scheme based on measured s.u.'s *w* = 1/(σ^2^(*I*) + 0.0004*I*^2^)
*S* = 1.63	(Δ/σ)_max_ = 0.034
1687 reflections	Δρ_max_ = 0.64 e Å^−^^3^
56 parameters	Δρ_min_ = −0.65 e Å^−^^3^
0 restraints	Absolute structure: 652 of Friedel pairs used in the refinement
35 constraints	Absolute structure parameter: 0.10 (9)

### Poly[(µ5-propanoato)caesium(I)] (IV) Fractional atomic coordinates and isotropic or equivalent isotropic displacement parameters (Å^2^)

**Table:**

	*x*	*y*	*z*	*U*_iso_*/*U*_eq_	Occ. (<1)
Cs1	0.73026 (5)	0.64992 (4)	0.191271 (13)	0.04419 (9)	
O1	0.7580 (11)	0.4754 (5)	0.32881 (17)	0.0553 (11)	
O2	0.7650 (9)	0.8283 (5)	0.32818 (19)	0.0571 (11)	
C1	0.7947 (8)	0.6530 (7)	0.3549 (2)	0.0406 (11)	
C2	0.891 (2)	0.6550 (12)	0.4209 (3)	0.095 (3)	
H1c2	0.793721	0.771881	0.442428	0.1141*	
H2c2	1.0977	0.706457	0.423612	0.1141*	
C3	0.861 (3)	0.4595 (15)	0.4580 (3)	0.099 (3)	
H1c3	0.943319	0.482452	0.498866	0.1485*	0.5
H2c3	0.650616	0.422829	0.461552	0.1485*	0.5
H3c3	0.967549	0.345746	0.437925	0.1485*	0.5
H1c3d	0.833861	0.340146	0.430661	0.1485*	0.5
H2c3d	1.039365	0.439114	0.482675	0.1485*	0.5
H3c3d	0.688258	0.471768	0.485008	0.1485*	0.5

### Poly[(µ5-propanoato)caesium(I)] (IV) Atomic displacement parameters (Å^2^)

**Table:**

	*U*^11^	*U*^22^	*U*^33^	*U*^12^	*U*^13^	*U*^23^
Cs1	0.04135 (14)	0.02910 (13)	0.06212 (18)	0.00031 (11)	−0.00274 (13)	0.00140 (11)
O1	0.065 (2)	0.0295 (15)	0.0712 (19)	−0.0046 (16)	−0.007 (2)	−0.0016 (13)
O2	0.061 (2)	0.0317 (15)	0.079 (2)	0.0006 (18)	−0.0072 (18)	0.0051 (14)
C1	0.0336 (18)	0.0278 (16)	0.060 (2)	0.0039 (19)	−0.0030 (16)	0.0009 (18)
C2	0.147 (7)	0.059 (4)	0.079 (5)	−0.009 (5)	−0.030 (5)	0.001 (4)
C3	0.147 (8)	0.089 (5)	0.061 (4)	0.013 (6)	−0.008 (5)	0.019 (4)

### Poly[(µ5-propanoato)caesium(I)] (IV) Geometric parameters (Å, º)

**Table:**

Cs1—O1	3.149 (4)	C2—H2c2	0.97
Cs1—O1^i^	3.006 (4)	C2—C3	1.471 (11)
Cs1—O1^ii^	3.082 (4)	H1c2—H2c2	1.4631
Cs1—O2	3.146 (4)	C3—H1c3	0.96
Cs1—O2^iii^	3.010 (4)	C3—H2c3	0.96
Cs1—O2^iv^	3.041 (4)	C3—H3c3	0.96
O1—C1	1.260 (5)	C3—H1c3d	0.96
O2—C1	1.249 (5)	C3—H2c3d	0.96
C1—C2	1.477 (9)	C3—H3c3d	0.96
C2—H1c2	0.97		
			
O1—Cs1—O1^i^	113.53 (11)	O1—C1—O2	124.3 (4)
O1—Cs1—O1^ii^	109.49 (11)	O1—C1—C2	118.0 (5)
O1—Cs1—O2	41.28 (8)	O2—C1—C2	117.6 (5)
O1—Cs1—O2^iii^	85.64 (11)	C1—C2—H1c2	109.47
O1—Cs1—O2^iv^	82.42 (11)	C1—C2—H2c2	109.47
O1^i^—Cs1—O1^ii^	93.22 (11)	C1—C2—C3	119.0 (6)
O1^i^—Cs1—O2	85.76 (9)	H1c2—C2—H2c2	97.9
O1^i^—Cs1—O2^iii^	85.08 (10)	H1c2—C2—C3	109.47
O1^i^—Cs1—O2^iv^	163.83 (10)	H2c2—C2—C3	109.47
O1^ii^—Cs1—O2	81.83 (9)	C2—C3—H1c3	109.47
O1^ii^—Cs1—O2^iii^	164.00 (10)	C2—C3—H2c3	109.47
O1^ii^—Cs1—O2^iv^	83.26 (10)	C2—C3—H3c3	109.47
O2—Cs1—O2^iii^	113.84 (10)	C2—C3—H1c3d	109.47
O2—Cs1—O2^iv^	109.21 (10)	C2—C3—H2c3d	109.47
O2^iii^—Cs1—O2^iv^	93.95 (9)	C2—C3—H3c3d	109.47
Cs1—O1—Cs1^iii^	94.31 (11)	H1c3—C3—H2c3	109.47
Cs1—O1—Cs1^iv^	97.42 (11)	H1c3—C3—H3c3	109.47
Cs1^iii^—O1—Cs1^iv^	93.22 (9)	H2c3—C3—H3c3	109.47
Cs1—O2—Cs1^i^	94.28 (11)	H1c3d—C3—H2c3d	109.47
Cs1—O2—Cs1^ii^	98.33 (11)	H1c3d—C3—H3c3d	109.47
Cs1^i^—O2—Cs1^ii^	93.95 (9)	H2c3d—C3—H3c3d	109.47
			
O1—C1—C2—C3	−16.7 (10)	O2—C1—C2—C3	165.7 (7)

Symmetry codes: (i) −*x*+1, *y*+1/2, −*z*+1/2; (ii) −*x*+2, *y*+1/2, −*z*+1/2; (iii) −*x*+1, *y*−1/2, −*z*+1/2; (iv) −*x*+2, *y*−1/2, −*z*+1/2.
